# Lowering effects of aspirin eugenol ester on blood lipids in rats with high fat diet

**DOI:** 10.1186/s12944-016-0369-2

**Published:** 2016-11-17

**Authors:** Isam Karam, Ning Ma, Xi-Wang Liu, Xiao-Jun Kong, Xiao-Le Zhao, Ya-Jun Yang, Jian-Yong Li

**Affiliations:** Key Lab of New Animal Drug Project of Gansu Province, Key Lab of Veterinary Pharmaceutical Development, Ministry of Agriculture, Lanzhou Institute of Husbandry and Pharmaceutical Science of CAAS, No.335, jiangouyan, qilihe district, Lanzhou, 730050 China

**Keywords:** Aspirin eugenol ester (AEE), Blood lipids, Body weight, High fat diet, Rats

## Abstract

**Background:**

Aspirin and eugenol were esterified to synthesize aspirin eugenol ester (AEE). As a pale yellow and odourless crystal, AEE reduced the gastrointestinal damage of aspirin and vulnerability of eugenol. The study was conducted to evaluate the preventive effects of AEE on blood lipids in rats with high fat diet (HFD).

**Methods:**

Suspensions of AEE and simvastatin were prepared in 5% carboxymethyl cellulose sodium (CMC-Na). In order to observe the intervention effects, the drugs and HFD were administrated at the same time. Based on individual weekly body weight (BW), AEE was intragastrically administrated at the dosage of 18, 36 and 54 mg/kg. Simvastatin (10 mg/kg) and CMC-Na (20 mg/kg) were used as control drug. After 6 weeks of administration, the changes of BW and blood lipid indices including triglyceride (TG), low density lipoprotein (LDL), high density lipoprotein (HDL) and total cholesterol (TCH) were determined in the experiment.

**Results:**

The rat blood lipids profile in model group was remarkably different after feeding 6-weeks HFD. TG, TCH and LDL indexes in model group were increased significantly compared with those in control group (*p* < 0.01). AEE at the dosage of 54 mg/kg significantly decreased levels of TG, TCH and LDL (*p* < 0.01), and slowed the rate of BW gain in comparison with model group (*p* < 0.05). Moreover, high dose AEE showed better effects than simvastatin on reducing TCH level and similar effects on TG, HDL and LDL.

**Conclusion:**

AEE could remarkably reduce levels of TG, TCH and LDL in rats with high fat diet, and slow the rate of body weight gain. It was conducted that AEE was a potential candidate on reducing blood lipids level. The mechanism of action of AEE should be investigated in further studies.

## Background

Hyperlipidemia is a heterogeneous group of disorders characterized by an excess of lipids in blood stream such as the increased serum levels of triglycerides (TG), total cholesterol (TCH), low-density lipoprotein (LDL) as well as decreased levels of high-density lipoprotein (HDL) [1, 2]. Hyperlipidemia, as the major risk factor for the development of cardiovascular diseases, is becoming a major health problem in the world [3].

Aspirin could ameliorate hyperlipidemia induced by high fat diet and hyperinsulinemia in rats [4]. Moreover, aspirin could diminish hypertriglyceridemia in obese rodents and has potential in hyperlipidemia prevention [5]. Eugenol as volatile oil is extracted from dry alabastrum of *Eugenia caryophyllata*. Therapeutic effects of eugenol on hyperlipidemia had been proved in previous study [6, 7]. Four-week administration of eugenol could significantly decrease the serum lipid profile in normal albino rabbits [8].

Based on prodrug principle and therapeutic effects of aspirin and eugenol on hyperlipidemia, aspirin eugenol ester (AEE) as a new drug was synthesized [9]. The metabolites of AEE had been confirmed in beagle dog and liver microsomes. AEE could be metabolized into aspirin and eugenol *in vitro* and *in vivo*, which could show their original activities and act synergistically [10]. AEE also reduced the side effects of its precursor such as the gastrointestinal damage of aspirin and irritation of eugenol [11]. The acute toxicity of AEE was less than its precursor, which was 0.02 times of aspirin and 0.27 times of eugenol [9, 11]. The teratogenicity and mutagenicity of AEE have been investigated. AEE did not show any mutagenesis in Ames test and the mouse bone marrow micronucleus assay [11, 12]. Moreover, the effects of AEE had been evaluated in animal model. The results showed that AEE had positive effects on antithrombosis, anti-inflammatory, analgesia and antipyretic [9, 11, 13].

Therapeutic strategies for hyperlipidemia treatment depend on reducing blood lipids. There are many chemical drugs that lower cholesterol level in the body such as statins, fibrates, ezetimibe and nicotinic acid. However, most of them are expensive and have undesirable effects [14]. There is an obvious need for more efficacious and alternative treatment options for hyperlipidemia. In our previous study, AEE (50 mg/kg and 160 mg/kg) could reduce TCH and TG in rats with standard diet [11]. Moreover, five-week administration of AEE (54 mg/kg) could normalize blood lipids profile in hyperlipidemic rats [15]. So there are increasing interest to evaluate the intervention effects of AEE on blood lipids in rat with high fat diet. This study will increase the understanding of AEE and provide impetus for further studies.

## Methods

### Chemicals and reagents

Aspirin eugenol ester (AEE), transparent crystal with the purity of 99.5% by RE-HPLC, was prepared in Key Lab of New Animal Drug Project of Gansu Province, Key Lab of Veterinary Pharmaceutical Development of Agricultural Ministry, Lanzhou Institute of Husbandry and Pharmaceutical Sciences of CAAS. CMC-Na and simvastatin was supplied by Tianjin Chemical Reagent Company (Tianjin, China). Standard compressed rat feed and high diet food were supplied by Keao Xieli Co., Ltd (Beijing, China). Standard rat diet consisted of 12.3% lipids, 63.3% carbohydrates, and 24.4% proteins (kcal) and high fat diet (standard rat diet 77.8%, yolk power 10%, lard 10%, cholesterol 2%, bile salts 0.2%) consisted of 41.5% lipids, 40.2% carbohydrates, and 18.3% proteins (kcal). The TG, TCH, LDL and HDL kits were provided by Ningbo Medical System Biotechnology Co., Ltd (Ningbo, China). Erba XL-640 analyzer (German) was used to measure the blood lipid indices.

### Animals

Seventy Sprague–Dawley (SD) male rats were purchased from the animal breeding facilities of Gansu University of Chinese Medicine (Lanzhou, China). The rats were housed in plastic cages of appropriate size (50 × 35 × 20 cm, ten rats per cage) with stainless steel wire cover and chopped bedding. Rat feed and drinking water were supplied *ad libitum*. Light/dark regime was 12/12 h and living temperature was 22 ± 2 °C with relative humidity of 55 ± 10%. Animals were acclimatized for 2 weeks before study initiation.

### Serum sampling

At the end of the experiment, rats were fasted for 10–12 h and then anaesthetized with 1% pentobarbital sodium. The blood samples were collected from the heart with vacuum tube. The sera were obtained by centrifuging for 15 mins at the speed of 4000 *g* at 4 °C. Serum samples were stored at −80 °C until the day of analysis.

### Drug preparation

AEE and simvastatin liquid suspensions were prepared in 0.5% of CMC-Na.

### Study design

Rats were randomly divided into seven groups including control, model, CMC-Na, simvastatin and three AEE groups. The detailed design of the experiment is shown in Table 1. Simvastatin was used as a positive control (10 mg/kg). The administrations of food and drugs were started at the same time. The volumes of CMC-Na and drug suspensions were nearly equal. After 6-weeks administration, the blood lipid levels were analyzed.

**Table 1:** Study design of the experiment

Groups	Food	Drug	Dosage	Concentration
Control	SRD	--	--	--
Model	HFD	--	--	--
CMC-Na	HFD	CMC-Na	20 mg/kg	5 mg/mL
Statin	HFD	Simvastatin	10 mg/kg	2.5 mg/mL
AEE Low	HFD	AEE	18 mg/kg	4.5 mg/mL
AEE Mid	HFD	AEE	36 mg/kg	9 mg/mL
AEE High	HFD	AEE	54 mg/kg	13.5 mg/mL

Rats were divided into seven groups. Control group was received SRD and the rest groups were received HFD, respectively. Based on the individual weekly body weight, rats were given with different drug suspension volume. CMC-Na as a vehicle was used in control group. Simvastatin was designed as positive drug. Different dose of three levels of AEE were administrated in the study. The administration period was 6 weeks and then blood lipids were analyzed

*AEE* aspirin eugenol ester, *SRD* standard rat diet, *HFD* high fat diet

### Statistics

The statistical analyses were carried out using IBM SPSS 19.0 (USA). All data obtained from the experiment are expressed as mean ± standard deviation (SD). Statistical differences were evaluated by using one-way ANOVA with Tukey’s multiple comparison tests. *P*-values less than 0.05 were considered statistically significant.

## Results

### Intervention effects of AEE

After feeding rats with HFD for 6 weeks, the blood lipid profile was notably different among the groups. There were significant differences between control and model groups (Table 2). In model group, TG, TCH and LDL were increased significantly in comparison with the control group (*p* < 0.01). However, no change was observed in HDL between control and model groups. There was no significant difference between CMC-Na and model groups. In comparing with model group, low and intermediate dose of AEE had no effect on blood lipid indexes except TCH index in AEE low group (*p* < 0.05). AEE high dose significantly decreased TG, TCH and LDL compared to model group (*p* < 0.01). AEE in different dosages had no influence on HDL. Simvastatin as a positive drug control could significantly decrease TCH and LDL (*p* < 0.01). With regard to TG and HDL, no changes were observed between simvastatin and model groups.

**Table 2:** The blood lipids levels after drugs administration for six weeks (*n* = 10)

Variables	Control	Model	CMC-Na	Statin	AEE Low	AEE Mid	AEE High
TG (mmol/L)	0.53 ± 0.12^**^	0.89 ± 0.07	0.79 ± 0.19	0.83 ± 0.05	0.79 ± 0.23	0.77 ± 0.10	0.71 ± 0.07^**^
HDL (mmol/L)	0.72 ± 0.04	0.74 ± 0.08	0.69 ± 0.09	0.66 ± 0.13	0.73 ± 0.09	0.79 ± 0.10	0.65 ± 0.15
LDL (mmol/L)	0.25 ± 0.02^**^	0.50 ± 0.09	0.46 ± 0.06	0.39 ± 0.13^**^	0.45 ± 0.05	0.46 ± 0.10	0.36 ± 0.08^**^
TCH (mmol/L)	1.4 ± 0.10^**^	2.29 ± 0.26	2.13 ± 0.22	1.80 ± 0.16^**^	2.10 ± 0.22^*^	2.15 ± 0.15	1.66 ± 0.15^**^

^*^ *P* < 0.05, ^**^ *P* < 0.01 significant difference compared to model group. *TG* triglyceride, *HDL* high density lipoprotein, *LDL* low density lipoprotein, *TCH* total cholesterol. Blood lipids indices were increased in model group. The blood lipid levels were reduced in simvastatin and high dose AEE groups in varying degrees

**Fig. 1:**
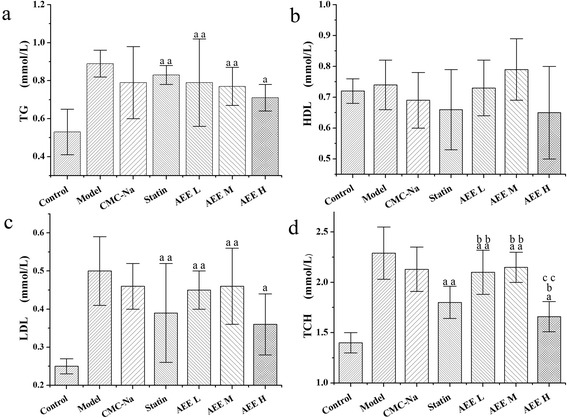
Comparative effects of AEE and simvastatin on blood lipid indexes (*n* = 10). ^a^ *P* < 0.05, ^aa^ *P* < 0.01 significant difference compared to control group; ^b^ *P* < 0.05, ^bb^ *P* < 0.01 significant difference compared to statin group; ^c^ *P* < 0.05, ^cc^ *P* < 0.01 significant difference compared to AEE L. CMC-Na: carboxymethylcellulose sodium, AEE L: low dose of AEE (18 mg/kg), AEE M: intermediate dose of AEE (36 mg/kg), AEE H: high dose of AEE (54 mg/kg). **a**, **b**, **c** and **d** were TG, HDL﻿, LDL and TCH, respectively

The three groups were used to compare with control and statin groups (Fig. 1). When compared with control group, TG, TCH and LDL in AEE groups were significant increased (*p* < 0.01 or *p* < 0.05). Notably, the mean values of TG, TCH and LDL in AEE high group were less than the values in low and intermediate AEE groups. Simvastatin and AEE made similar effects on TG, HDL and LDL indexes. However, simvastatin showed more positive effects on TCH than AEE L and AEE M but not AEE H (*p* < 0.01 or *p* < 0.05 Fig. 1d). In order to find out the relationship between drug effects and the dosage, intermediate and high AEE groups were compared with low dose AEE group. The results showed that there was only significant difference on TCH index in AEE high group (*p* < 0.01 Fig. 1d). Interestingly, the mean values of TG and LDL in AEE high group were the smallest in all AEE groups.

### Body weight

The body weights of all groups were recorded at the beginning and ending of the experiment (Table 3). At the beginning of the experiment, there was no significant difference between all groups. At week 6, the body weights in the group supplemented with AEE high dose were significantly lower than those in model group (*p* < 0.05). The results showed that high dose of AEE could reduce the rate of body weight gain. The body weights in AEE intermediate and high groups were remarkably lower than control group (*p* < 0.05 or *p* < 0.01). No difference was observed among statin and AEE groups. The gains in body weights in AEE high group were significantly lower than those in AEE low group (*p* < 0.05).

**Table 3:** Body weights of rats at the beginning and the end of the experiment (*n* = 10)

Groups	Week 1	Week 6
Control	209 ± 15	385 ± 20
Model	216 ± 11	368 ± 25
CMC-Na	209 ± 10	365 ± 20
Statin	220 ± 22	355 ± 20^a^
AEE Low	220 ± 17	366 ± 31
AEE Mid	211 ± 15	356 ± 20^a^
AEE High	213 ± 14	340 ± 15^*aab^

^*^ *P* < 0.05 significant difference compared to model group; ^a^ *P* < 0.05, ^aa^ *P* < 0.01 significant difference compared to control group; ^b^ *P* < 0.05 significant difference compared to AEE low group; Week 1: beginning of the experiment; Week 6: the end of the experiment

## Discussion

Based on prodrug principle, aspirin and eugenol, as starting precursors, were esterified to synthesize aspirin eugenol ester (AEE). The positive effects of AEE on the symptoms of inflammation, fever, pain and thrombosis had been confirmed in animal disease model [9, 11, 13]. The blood chemistry results in 15-day oral dose toxicity study showed AEE could significantly reduce the values of TG and TCH both in male and female rat, which indicated that this compound was potential for curing hyperlipidemia [11]. In subsequent experiment, the regulation effects of AEE on blood lipid profile in hyperlipidemic rats had been confirmed [15]. Based on this fact, it is essential to evaluate the intervention effects of AEE on blood lipids in rats with high fat diet fed.

The intervention effect of AEE was evaluated by comparing AEE groups with model group. AEE low and intermediate dose showed no significant effect on blood lipid indices except reducing TCH at AEE low dose. It is noteworthy that high dose of AEE was highly effective in reducing blood lipid indices such as TG and TCH. These meant that the activity of AEE at high dose was better than low and intermediate doses. So there was a positive relationship between drug efficacy and dosage. Under the present experimental conditions, the optimal dose was considered to be 54 mg/kg for normalizing blood lipid profile in high fat diet fed rat.

HDL is responsible for the transportation of cholesterol to liver, which is essential for cholesterol removal [16]. HDL levels of rat fed with high fat diet for six weeks showed no changes in this study. There are several possible reasons for this result. First of all, HFD compositions such as the lack of saturated fat may be insufficient to make substantial change on HDL. Second, the constant values of HDL may be attributable to the short duration of the experiment (only 6 weeks). Finally, animal model used in the experiment may be a potential cause. Literature indicated that most cholesterol in rat blood circulation existed in HDL and this contributed to reducing susceptibility of HDL level to HFD [17, 18].

HFD is a commonly used material to induce animal disease such as hyperlipidemia, diabetes, obesity and arteriosclerosis [19, 20]. In this experiment, the results of body weight were not consistent with those in our previous study. There was no difference in body weight between control and model group. However, HFD with same components significantly increased body weight at the end of eighth week in Wistar rat [15]. The shortage of HFD consumption time may be the reason for the results of no significant difference in body weight. Metabolism and liver function could be substantially changed with HFD feeding [21]. It was necessary to give the rat time to adjust for HFD gradually, which may slow the rate of the body weight gain in certain period. It was found that AEE dosage had different influence on body weight. AEE high and middle dose made an impact on body weight and there was significant difference between low and high AEE groups. The results of AEE on body weight were also the reason why 54 mg/kg was suggested for optimal dosage.

Simvastatin is an inhibiter of 3-hydroxy-3-methylglutaryl coenzyme A (HMG-CoA) which plays a crucial role in cholesterol synthesis in liver cells. Simvastatin was used as a positive drug control in this study and it decreased the levels of TCH and LDL. The effects of 10 mg/kg simvastatin on TCH and LDL were similar with 54 mg/kg AEE. However, simvastatin had no influence in TG index and showed significant difference from high dose AEE. From these results, it could be concluded that AEE was more effective than simvastatin on reducing blood lipid level. CMC-Na was widely used as a reliable drug vehicle in pharmaceutical industry [22, 23]. The effect of CMC-Na on blood lipid indexes was eliminated by administrating equal volume CMC-Na to control group. Therefore, it manifested that the effects of AEE was not related to CMC-Na.

Therefore, intragastrical administration of AEE had a significant intervention effects in HFD fed rat. AEE was decomposed into salicylic acid and eugenol after absorption. The effects of AEE on reducing blood lipid indexes may be mainly from synergetic action of aspirin and eugenol. More studies are necessary to investigate the action mechanism of AEE such as evaluation of inhibitory effect on digestive enzymes and influence on metabolic targets.

## Conclusions

Under the present study condition, AEE at daily dosage of 54 mg/kg BW for six weeks could remarkably reduce levels of TG, TCH and LDL in rats with high fat diet, and slow the rate of body weight gain. Moreover, AEE at 54 mg/kg showed better effects than simvastatin at 10 mg/kg on reducing TCH level and similar effects on TG, HDL and LDL.

## Abbreviations

AEE: Aspirin eugenol ester
BW: Body weight
CMC-Na: Sodium carboxymethyl cellulose
HDL: High density lipoprotein
HFD: High fat diet
LDL: Low density lipoprotein
SRD: Standard rat diet
TCH: Total cholesterol
TG: Triglycerides
